# Apoidea of the collections of Lyon, Aix-en-Provence, Marseille and Toulon Museums of Natural History (France)

**DOI:** 10.3897/BDJ.11.e99650

**Published:** 2023-03-13

**Authors:** Jean-Yves Meunier, Benoît Geslin, Mehdi Issertes, Gilles Mahé, Frédéric Vyghen, Harold Labrique, Yves Dutour, Vincent Poncet, Jérémy Migliore, Gabriel Nève

**Affiliations:** 1 IMBE, Marseille, France IMBE Marseille France; 2 IRD, Marseille, France IRD Marseille France; 3 CNRS, Marseille, France CNRS Marseille France; 4 Avignon Université, Marseille, France Avignon Université Marseille France; 5 Aix-Marseille Université, Marseille, France Aix-Marseille Université Marseille France; 6 Observatoire des Abeilles, Flines-lez-Raches, France Observatoire des Abeilles Flines-lez-Raches France; 7 Unaffiliated, Mesquer, France Unaffiliated Mesquer France; 8 Arthropologia, La-Tour-de-Salvagny, France Arthropologia La-Tour-de-Salvagny France; 9 Musée des Confluences, Lyon, France Musée des Confluences Lyon France; 10 Muséum d’Histoire Naturelle, Aix-en-Provence, France Muséum d’Histoire Naturelle Aix-en-Provence France; 11 Muséum d’Histoire Naturelle, Marseille, France Muséum d’Histoire Naturelle Marseille France; 12 Muséum départemental du Var, Toulon, France Muséum départemental du Var Toulon France

**Keywords:** Hymenoptera, bees, museum, France, record, Apoidea, *Bombus*, Apidae, Halictidae, Andrenidae, Megachilidae, Colletidae, Melittidae

## Abstract

**Background:**

Many insect species have shown dramatic declines over the last decades, as a result of man-related environmental changes. Many species which were formerly widespread are now rare. To document this trend with evidence, old records of collected specimens are vital.

**New information:**

We provide here the data on 9752 bee (Hymenoptera: Apoidea) specimens hosted in several museums of south-east France: Musée des Confluences in Lyon, Muséum d’Histoire Naturelle de Marseille, Muséum d’Aix-en-Provence and the Muséum Départemental du Var in Toulon. Most of the specimens (9256) come from France and include data on 552 named species. For most of these specimens, the geographical location, including geographical coordinates, is based on the locality (town or village) where they were collected. The specimens were captured from the beginning of the nineteenth century to 2018. The identifications of 1377 specimens, mainly belonging to the genus *Bombus*, are considered reliable, as these were performed or been checked since 2009. All the other reported identifications are the original ones given by the original collectors.

## Introduction

There has been a dramatic decline in insect populations over the last 70 years, both in terms of abundance (Hallmann et al. 2017, Seibold et al. 2019) and diversity (Raven and Wagner 2021). To document the past occurrences of species in areas where they have now decreased or vanished, entomologists mostly rely on material preserved in collections (e.g. Decker et al. (2020), Mathiasson and Rehan (2020)). Museum materials are what remains of past ecosystems; for some species, they represent the last testimony of their presence in a region or country and it appears crucial to extract as much information as possible from these specimens (Raven and Miller 2020), such as photographs of key specimens. In many natural history museums around the world, there are thousands of specimens stored in collections for which we barely have any information. This is particularly the case for insects, which are difficult to preserve because of pests and difficult to identify due to the taxonomic impediment (Engel et al. 2021). Yet, the importance of insects in the functioning of ecosystems should urge precise monitoring of museum collections contents. In the present time of rapid anthropogenic ecological changes at all scales, we must find ways to preserve these specimens as well as we can for as long as we can. They are vulnerable to degradation and loss from pests (Verlinden 2020), humidity (Clary 1991), fire (Escobar 2018) and the toll of time. While renewing efforts to protect them, we need to make them more accessible through digitisation, including imaging (Paterson et al. 2016) and, for a representative series of specimens, the sequencing of COI and other genes.

Amongst the various roles of insects in ecosystems, the pollination process is a key component, both in natural and man-made ecosystems (Hristov et al. 2020). Amongst pollinators, Hymenoptera is often considered as the most important insect order to pollinate flowers, followed by Diptera and Lepidoptera (Walton et al. 2020). Amongst Hymenoptera, Anthophila (bees *sensu lato*) is the main group of pollinators. The French bee fauna currently includes 978 species (Ropars, pers. comm.). Work is currently in progress both in revising the taxonomy of species groups which are not covered by recent reliable keys (e.g. Le Divelec (2021) on *Epeolus* genus) or in regional lists (e.g. Terret et al. (2020) for Franche-Comté). Despite these recent revisions, the available information about the diversity of bees within the French territory, both current and past, is still very incomplete. The design and implementation of effective conservation measures rely on the knowledge of both current and historic distribution of species (Schatz et al. 2021), which in turn, relies on the knowledge on the bee species distribution within the French territory.

The aim of the present paper is to document the collections of Apoidea hosted by the Musée des Confluences in Lyon (MHNL), the Muséum d’Histoire Naturelle de Marseille (MHNM), the Muséum d’Aix-en-Provence (MHNAix) and the Muséum Départemental du Var (MDV) in Toulon (Table 1). The Musée des Confluences holds the second most important Natural history collection in France, after the Paris Museum National d’Histoire Naturelle. It was founded in 1772 (Clary 1995). The Marseille Museum, founded in 1819, currently holds a collection of ca. 84,000 zoological specimens (Lima and Médard 2021). The Aix Museum was founded in 1838 (Sepulveda and Dutour 2006) and hosts an important collection of insects collected in the vicinity of Aix-en-Provence in the nineteenth century (Dusoulier 2006).

Ultimately, the knowledge of past occurrences of bees will be of outmost importance for documenting the evolution of bee richness and their distribution in France and to set the basis for a future French Red List of bees which still does not exist to this day.

## General description

### Purpose

The aim of this publication is to make public the records of Apoidea stored in the four natural history Museums of south-east France. Researchers will, therefore, know where the specimens are stored, how numerous they are and when and where they were collected. Information on who identified the specimens and when is also given, as this is important given the on-going changes in the taxonomic treatment of many species (Rasmont et al. 2017, Gargominy et al. 2021). The past status of species now rare may then be assessed at a regional level and the original specimens may then be localised, studied and re-identified if needed.

## Project description

### Title

Apoidea collections in the natural history museums of south-east France.

## Sampling methods

### Study extent

All available data on the Apoidea specimens stored in the four natural history Museums were input into a table format. Most original labels include location (usually the municipality), date (sometimes only partly, such as the month), collector and an assigned species name. As the taxonomy of the Apoidea has dramatically changed over the last 150 years, we believe that an important part of the old material should be re-identified according to current knowledge. This could be done only for a part of the specimens: Bumblebees (*Bombus*) in the Musée des Confluences were recently revised by MI, GM and FV and all specimens at the MDV were identified since 2009; their *identificationVerificationStatus* column was coded “1”. The data on all the other specimens have retained their original species identification and the *identificationVerificationStatus* column was coded “0”.

The collectors of Apoidea specimens in the four Museums were: René Grilat (?-1915) [2645 specimens], Georges Audras (1881 -1970) [2028 specimens], Maurice Dauzet (1927-2021) [1752 specimens], Claudius Côte (1881-1956) [821 specimens], Jean Timon-David (1902-1968) [606 specimens], Pierre Réal (1922-2009) [392 specimens], Jean-Hubert Chabrier (1791-1884) [240 specimens], Claude Dufay (1926-2001) [133 specimens], Philippe Grivot [131 specimens], Nicolas Bermante [128 specimens], Jacques Hamon (1926-2022) [56 specimens], Guy Chavanon (born 1951) [78 specimens], Robert Gonon (1908-1994) [53 specimens], Roland Allemand (1950-2013) [29 specimens] and several others.

### Sampling description

On top of the data given on the original labels, we have added the Department (French administrative division) and the coordinates of the centre of the locality where each specimen was collected (columns *decimalLatitude* and *decimalLongitude*). This gives an approximation of ca. 5 km, depending on each locality size.

### Quality control

The specialists whose recent identifications we relied on are Holger Dathe (genus *Hylaeus*), Robert Fonfria (mainly Families Megachilidae and Andrenidae), David Genoud (genus *Andrena*), Michael Kuhlmann (genus *Colletes*), Gérard Le Goff (genus Anthophora), Hugues Mouret (genera *Anthophora* and *Andrena*), Alain Pauly (family Halictidae), Stephan Risch (genus *Eucera*) and Erwin Scheuchl (genus *Andrena*) for the 89 specimens in the MDV collections and MI, GM and FV for 1288 *Bombus* specimens in MHNL.

### Step description

The collections of Apoidea in the Natural History Museums of Aix-en-Provence, Lyon, Marseille and Toulon were surveyed systematically. A total of 9752 specimens were recorded. A total of 1377 French specimens were either checked in MHNL or identified recently by various specialists, as in the case of the specimens from Porquerolles (Hyères, Var) in MDV.

For all other specimens, the nomenclature was checked against the list of European bees used for the IUCN Red List (Nieto et al. 2014). If the original species name did not appear in this reference list, a search was done on  http://westpalbees.myspecies.info  and  https://www.bwars.com/search/node/Caelioxis  websites to find the name currently used for names recognised as synonyms. The current name was, thus, mentioned in the *scientificName* column, whereas the name given by the original identifier was put into the *previousIdentifications* column. If the original name could not be assigned unambiguously to a current name or if no identification had been given, no current species name was given in the data set.

As far as possible, the locality of origin of the specimen was identified and its latitude and longitude given by the website https://www.geoportail.gouv.fr/ was input. In a few cases, such as passes or forests between neighbouring localities, the precise coordinates of the location were input. In the CSV dataset format, fields are separated by tabs, all encoding is UTF-8, which allowed for all diacritic signs to be retained. Apostrophes (') were used wherever appropriate in locality names. Uncertain readings from the labels are indicated by a question mark in the *verbatimEventDate* or *verbatimLocality* fields. If the locality name was uncertain, no coordinates were given.

## Geographic coverage

### Description

**Figure 1.**
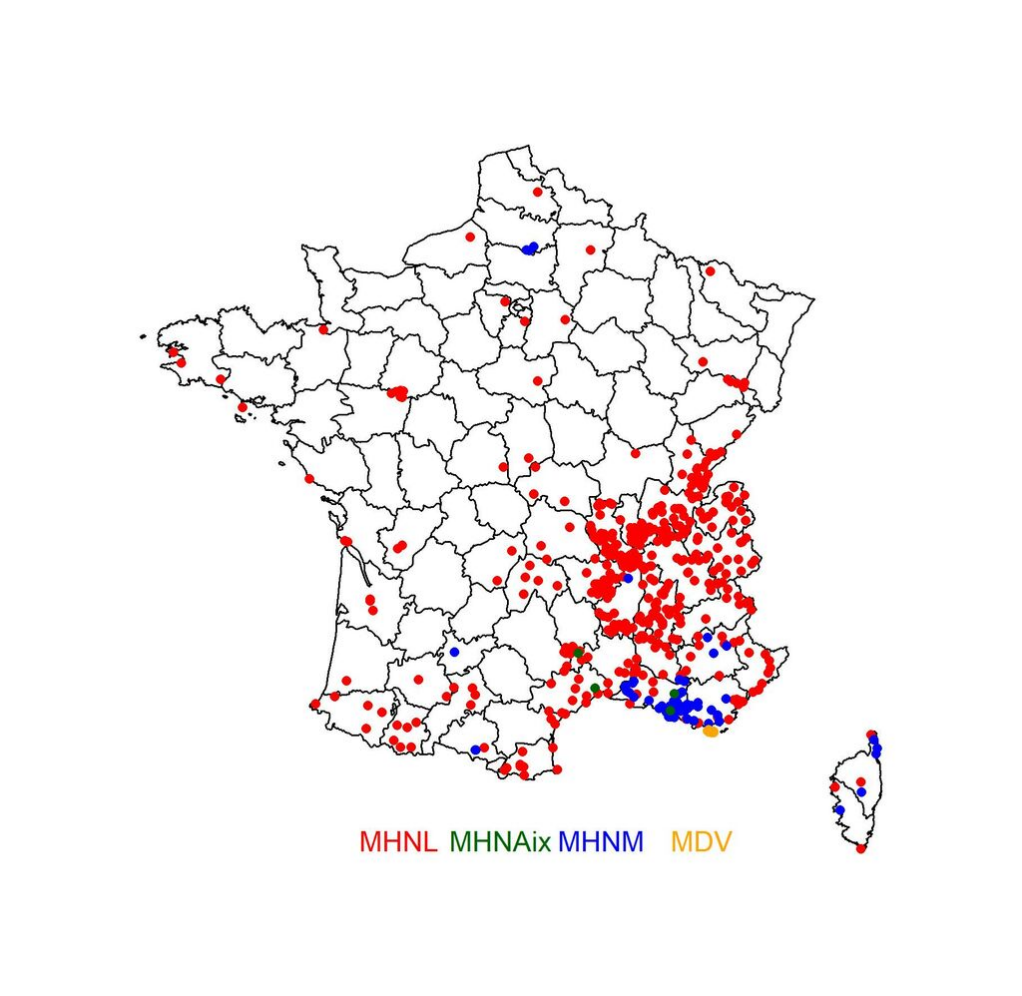
Geographical distribution of the surveyed specimens in France, according to the holding Museums.

The Apoidea specimens mainly come from south-east France (Fig. 1), but also include specimens from 24 other countries: Algeria [10], Austria [6], Brazil [2], Chad [1], Czech Republic [46], Germany [7], Greece [10], Guatemala [1], Hungary [3], Indonesia [1], Italy [4], Ivory Coast [2], Luxembourg [2], Morocco [8], The Netherlands [31], Romania [1], Slovakia [2], Spain [8], Switzerland [6], Tunisia [15], Turkey [9], United Kingdom [22], USA [1] and former Yugoslavia [2]. The country of origin of 296 specimens could not be traced. The 9256 specimens from France come from 61 Departments (Table 2), mainly Rhône (4253 specimens), Loire (1141 specimens), Bouches-du-Rhône (874 specimens) and Ain (554 specimens). The localities of 139 specimens could not be traced to a Department.

### Coordinates

41 and 51 Latitude; 10 and -5 Longitude.

## Taxonomic coverage

### Description

Specimens of at least 552 species are present in the collections of the four surveyed natural history Museums. The specimens belong to the families Apidae [3153 specimens], Halictidae [1866 specimens], Andrenidae [1597 specimens], Megachilidae [1092 specimens], Colletidae [527 specimens] and Melittidae [52 specimens]. Forty-one genera have been identified; the genera *Bombus* and *Andrena* are present with more than a thousand specimens each (Table 3). Only eleven species are represented by at least 50 specimens in the collections of the four natural history Museums (Table 4).

### Taxa included

**Table:** 

Rank	Scientific Name	
superfamily	Apoidea	
family	Apidae	
family	Halictidae	
family	Andrenidae	
family	Megachilidae	
family	Colletidae	
family	Melittidae	

## Temporal coverage

**Data range:** 1801-1-01 – 2018-9-05.

### Notes

**Figure 2.**
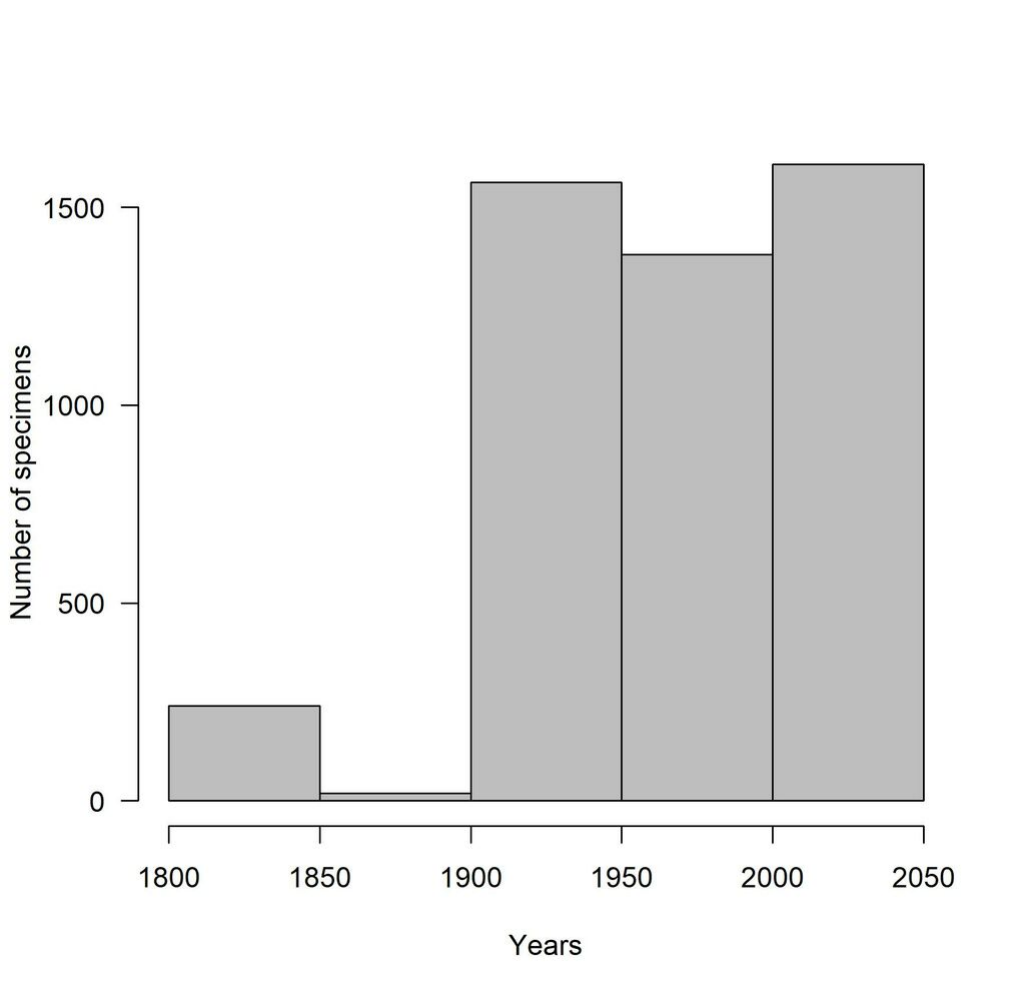
Periods of captures of surveyed Apoidea specimens in the four Museums.

The oldest specimens are those collected by Jean-Hubert Chabrier (1791-1884), hosted in MHNAix, which presumably come mostly from the first half of the nineteenth century, but do not bear any date information (Dusoulier 2006). The most recent specimens are those of Maurice Dauzet (1927-2021) who collected until 2018 and later donated his collection to MNHL. The historic distribution of the data shows that, apart from the 250 specimens from Chabrier’s collection, most of the specimens come from the 20^th^ century and the first 20 years of the 21^st^ century (Fig. 2).

## Usage licence

### Usage licence

Creative Commons Public Domain Waiver (CC-Zero)

### IP rights notes

This work is licensed under a Creative Commons Attribution (CC-BY) 4.0 Licence. All work derived from the present study should cite it appropriately, including the Museum where the material is held.

## Data resources

### Data package title

Apoidea at the Lyon, Marseille, Aix-en-Provence and Toulon Museums

### Resource link

https://doi.org/10.5281/zenodo.7456986

### Number of data sets

1

### Data set 1.

#### Data set name

Apoidea at four Museums of SE France: Apoidea_data_SE_France.csv

#### Data format

CSV (tab delimited values)

#### Download URL

https://doi.org/10.5281/zenodo.7456986

#### Data format version

Darwin core, so that it could be transferred later into GBIF as the identifications are checked and more precise locations entered.

#### Description

The whole dataset includes 9752 Apoidea specimens from the Muséum d’Histoire Naturelle d’Aix en Provence (MHNAix), the Musée des Confluences, Lyon (MHNL), the Muséum d’Histoire Naturelle de Marseille (MHNM) and the Muséum Départemental du Var, Toulon (MDV). This dataset uncludes 1377 specimens with a recent reliable identification and 9256 with geolocalisation within France (Table 1).

**Data set 1.** 

Column label	Column description
occurrenceID	Individual identification: combination of Museum name, collection identification, box number and specimen number within each box.
basisOfRecord	The specific nature of the data record (i.e. PreservedSpecimen).
eventDate	Event date in the format YYYY-MM-DD if the date is known to the day, or YYYY-MM if only the month and the year are known, or YYYY if only the year is known.
Year	Year of capture if known.
Month	Month of capture if known.
Day	Day of capture if known.
verbatimEventDate	Date of capture, if known, in format DD/MM/YYYY. Missing data are indicated by ?
scientificName	Lowest taxonomic rank possible, usually the species name. If the species is unknown, the genus or family names are given.
Kingdom	Kingdom (i.e. Animalia).
Phylum	Phylum (i.e. Arthropoda).
Class	Class (i.e. Insecta).
Order	Order (i.e. Hymenoptera).
family	Family name.
genus	Genus name.
specificEpithet	Species epithet of the scientificName.
sex	Male (M) or Female (F).
taxonRank	Taxonomic rank of the most specific name in the scientificName.
IdentifiedBy	Name of the entomologist who identified the specimen, if indicated by the label.
dateIdentified	Year of identification, if known.
identificationVerificationStatus	Whether (coded 1) or not (coded 0) the identification was recently (since 2009) checked.
decimalLatitude	Geographic latitude (in decimal degrees) of the location.
decimalLongitude	Geographic longitude (in decimal degrees) of the location.
geodeticDatum	Coordinate system and set of reference points upon which the geographic coordinates are based (i.e. WGS 84).
coordinateUncertaintyInMeters	Uncertainty in coordinates. As the coordinates are usually those of the locality of the record, uncertainty is in the range of 5000 m.
Country	Country of capture, in French, as indicated by the label.
countryCode	Two letter country code of the specimen origin.
stateProvince	French departmental administrative division. In the case of non-French data, any relevant country administrative subdivision.
locality	Location of capture, usually the locality.
verbatimLocality	Any geographical indication on the label.
InstitutionCode	Museum where the specimen is held.
CatalogNumber	Box identifier within each Museum.
occurrenceRemarks	Any ecological data or comment on the label
recordedBy	Name of collector (i.e. *legit* information).
OrganismQuantity	Number of individuals bearing the same label (usually 1).
OrganismQuantityType	individuals.
previousIdentifications	Species name originally given by the original collector, if different from scientificName.
georeferencedBy	Identity of the person who added the Latitude and longitude data, usually Meunier, Jean-Yves.
georeferenceProtocol	How the georeference was computed, i.e. from label data (verbatimLocality).
georeferenceSources	Georeference code was inferred from geoportail.fr.
georeferencedDate	Georeference work was mainly performed in 2021, with a few additions in 2023.
language	The data set is mainly written in French, apart from column headings, which are in English
CollectionCode	Identifier of collection within each Institution where specimens are held.
locationRemarks	Several localities could not be identified unambiguously, this is indicated by “localité incertaine” in this field.

## Additional information

### Specimen preservation methods

Dried and pinned specimens.

### Abbreviations used throughout

MHNAix: Muséum d’Histoire Naturelle d’Aix en Provence (Bouches-du-Rhône)

MHNL: Musée des Confluences, Lyon (Rhône)

MHNM: Muséum d’Histoire Naturelle de Marseille (Bouches-du-Rhône)

MDV: Muséum Départemental du Var, Toulon (Var)

### Publishing organisations

Musée des Confluences, Lyon (MHNL)

Muséum d’Histoire Naturelle de Marseille (MHNM)

Muséum d’Histoire Naturelle d’Aix-en-Provence (MHNAix)

Muséum Départemental du Var, Toulon (MDV)

### Museum identifiers

MHNL, MHNM, MHNAix, MDV.

### Contacts

MHNL: Harold Labrique: harold.labrique@museedesconfluences.fr

MHNM: Vincent Poncet: vponcet@marseille.fr

MHNAix: Yves Dutour: geologie_aix@yahoo.fr

MDV: Jérémy Migliore: jmigliore@var.fr

**dataset management**

Gabriel Nève: gabriel.neve@imbe.fr

### General discussion

Altogether, the studied collections hold a total of 9752 Apoidea specimens at the time of writing. A total of 9256 specimens are from mainland France or Corsica (Fig. 1) and 295 specimens have no locality information. All the following analyses are based on mainland France and Corsica data only.

Unfortunately, 5002 of the 9255 French specimens do not bear a date of collection. For some of these, the time frame was guessed using the biographic data of the collectors. If we hypothesise that the specimens from the Chabrier Collection were collected during the first half of the 19^th^ century and the ones from Côte Collection during the first half of the 20^th^ century, most of the specimens were collected since 1900, equally divided (about 1500 specimens) in each of the time spans 1900-1949, 1950-1999 and 2000-2018 (Fig. 2). The 4253 specimens with accurate collection data (day, month, year) date from 1881 to 2018.

**Figure 3.**
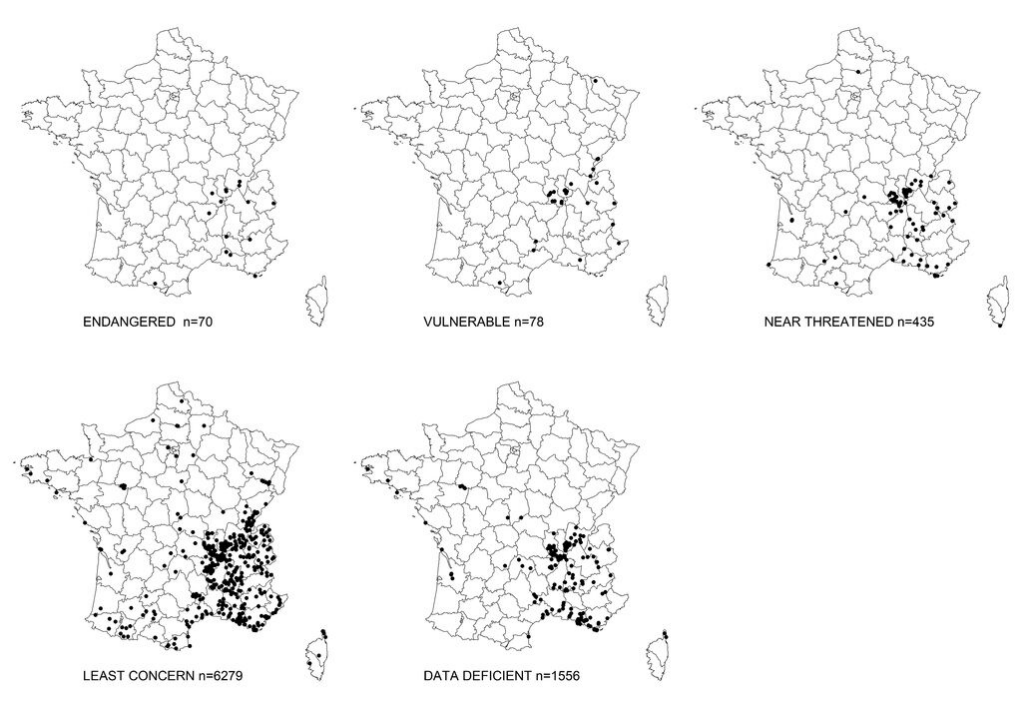
Distributions of Apoidea specimens according to European IUCN criteria.

**Figure 4.**
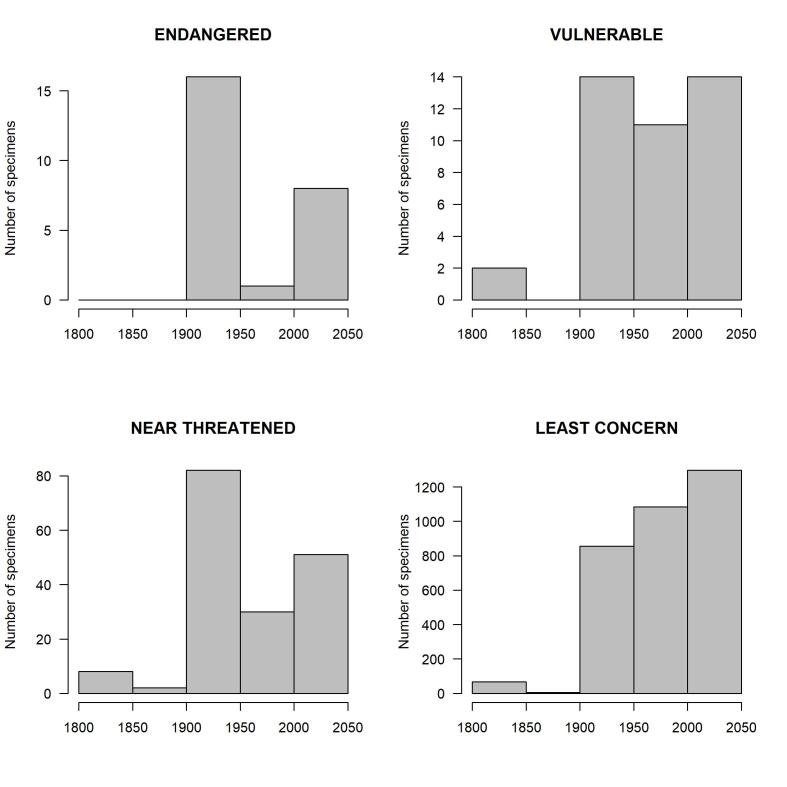
Period of capture according to the European IUCN criteria for all surveyed Apoidea specimens.

The temporal distribution of the data according to the IUCN criteria of the European fauna (Nieto et al. 2014) shows that most specimens belonging to endangered species were collected either in the years 1901-1950, or in the years 2000-2018 (Fig. 3, Fig. 4). Only one specimen, captured between 1950 and 2000, belonged to an endangered species (*Trachusainterrupta*), whereas a total of eight specimens of endangered species have been collected since 2000 (Table 5). On the other hand, four endangered species have no data since 1950: *Lasioglossumquadrisignatum*, *Lasioglossumsubfasciatum*, *Melittamelanura* and *Osmiamaritima*, leaving the question open as to whether they still occur in France. Altogether, the dataset holds data on ten species listed as endangered (Table 5) and eight species classified as vulnerable in Europe (Table 6).

From the recently checked 1301 *Bombus* specimens from MHNL, 713 did not bear any previous identification label at the species level. Amongst the 588 *Bombus* specimens bearing identification labels, 362 (62%) had an identification label which matched the recent species check; all the other specimens had their original identification corrected. This underlines the need for experts to check Museum collections in order to validate their data. The work of presenting the basic data allows the experts to know how many specimens there are in the surveyed Museums and also when and where the specimens come from.

## Figures and Tables

**Table 1.** Number of recorded French Apoidea specimens by Museum.

**Museum acronyms**	**Museum Full Names**	**Total number of specimens**	**Number of specimens from France**	**Number of French geolocalised specimens**	**Number of specimens identified since 2000**
**MHNAix**	Muséum d'Histoire Naturelle d'Aix-en-Provence	255	243	153	0
**MHNL**	Musée des Confluences (Lyon)	8790	8312	8239	2646
**MHNM**	Muséum d'Histoire Naturelle de Marseille	612	606	589	0
**MDV**	Muséum Départemental du Var (Toulon)	95	95	95	95
**Total**		9752	9256	9076	2741

**Table 2.** Numbers of French Apoidea specimens, sorted by French Departments.

Departments	N
Ain	554
Aisne	1
Allier	4
Alpes-de-Haute-Provence	29
Alpes-Maritimes	16
Ardèche	80
Ariège	28
Aude	5
Aveyron	4
Bouches-du-Rhône	874
Cantal	5
Charente	4
Charente-Maritime	10
Cher	1
Corrèze	24
Corse	14
Corse-du-Sud	10
Doubs	86
Drôme	296
Essonne	3
Finistère	18
Gard	73
Gers	14
Gironde	29
Guyane française	1
Haute-Corse	31
Haute-Garonne	15
Haute-Loire	308
Haute-Saône	64
Haute-Savoie	71
Hautes-Alpes	36
Hautes-Pyrénées	8
Hérault	76
Indre	2
Isère	219
Jura	155
Landes	3
Loire	1141
Loiret	2
Lozère	2
Manche	1
Morbihan	5
Moselle	1
Pas-de-Calais	1
Puy-de-Dôme	11
Pyrénées-Atlantiques	6
Pyrénées-Orientales	23
Rhône	4253
Saône-et-Loire	1
Sarthe	41
Savoie	114
Seine-et-Marne	1
Seine-Maritime	2
Somme	8
Tarn-et-Garonne	2
Territoire de Belfort	11
Var	243
Vaucluse	63
Vendée	7
Vosges	6
Yvelines	1
Unknown	139
TOTAL	9256

**Table 3.** Number of specimens per genus for all French specimens.

Genus	Number of specimens
*Amegilla*	4
*Ammobates*	4
*Andrena*	1056
*Anthidiellum*	6
*Anthidium*	124
*Anthophora*	193
*Apis*	15
*Biastes*	19
*Bombus*	1376
*Ceratina*	85
*Chelostoma*	49
*Coelioxys*	73
*Colletes*	138
*Dasypoda*	11
*Dioxys*	3
*Dufourea*	2
*Epeolus*	43
*Eucera*	80
*Halictus*	390
*Heriades*	4
*Hoplitis*	19
*Hylaeus*	94
*Icteranthidium*	2
*Lasioglossum*	227
*Lithurgus*	16
*Macropis*	2
*Megachile*	137
*Melecta*	29
*Melitta*	8
*Nomada*	521
*Osmia*	188
*Panurgus*	76
*Pasites*	6
*Rhodanthidium*	13
*Sphecodes*	205
*Stelis*	27
*Systropha*	10
*Tetralonia*	2
*Thyreus*	3
*Trachusa*	2
*Xylocopa*	74

**Table 4.** Species with more than 50 French specimens.

Species	Number of specimens
*Bombuslapidarius*	161
*Bombuslucorum*	141
*Bombuspascuorum*	141
*Apismellifera*	92
*Bombusterrestris*	79
*Bombuspratorum*	75
*Bombussylvestris*	62
*Bombussoroeensis*	56
*Andrenaflavipes*	53
*Halictusscabiosae*	52

**Table 5.** Numbers of French specimens of species classified as endangered in Europe, according to time-frames and Museums where held.

Time frame	Species	Museum	Number of specimens
1901-1950	*Lasioglossumlaeve*	MHNL	3
1901-1950	*Lasioglossumquadrisignatum*	MHNL	2
1901-1950	*Lasioglossumsubfasciatum*	MHNL	2
1901-1950	*Melittamelanura*	MHNL	3
1901-1950	*Trachusainterrupta*	MHNM	5
1901-1950	*Osmiamaritima*	MHNL	1
1951-2000	*Trachusainterrupta*	MHNL	1
2001-2018	*Colletescollaris*	MDV	2
2001-2018	*Lasioglossumbreviventre*	MHNL	2
2001-2018	*Lasioglossumlaeve*	MHNL	3
2001-2018	*Trachusainterrupta*	MHNL	1
unknown	*Halictuscarinthiacus*	MHNL	1
unknown	*Halictussemitectus*	MHNL	8
unknown	*Lasioglossumlaeve*	MHNL	4
unknown	*Lasioglossumquadrisignatum*	MHNL	16
unknown	*Lasioglossumsubfasciatum*	MHNL	7
unknown	*Melittamelanura*	MHNL	3
unknown	*Trachusainterrupta*	MHNL	6
Total			70

**Table 6.** Numbers of French specimens of species classified as vulnerable in Europe, according to time-frames and Museums where held.

Time frame	Species	Museum	Number of specimens
1801-1850	*Bombusmuscorum*	MHNAix	2
1901-1950	*Bombusconfusus*	MHNL	3
1901-1950	*Bombusdistinguendus*	MHNL	1
1901-1950	*Bombuspomorum*	MHNL	10
1951-2000	*Bombusalpinus*	MHNL	2
1951-2000	*Bombusconfusus*	MHNL	4
1951-2000	*Bombusdistinguendus*	MHNL	1
1951-2000	*Bombusgerstaeckeri*	MHNL	1
1951-2000	*Bombusmuscorum*	MHNL	3
2001-2018	*Bombusconfusus*	MHNL	6
2001-2018	*Bombusmuscorum*	MHNL	1
2001-2018	*Bombuspomorum*	MHNL	1
2001-2018	*Colletesflorealis*	MHNL	3
2001-2018	*Colletesfodiens*	MHNL	3
unknown	*Bombusconfusus*	MHNL	15
unknown	*Bombusdistinguendus*	MHNL	3
unknown	*Bombusmuscorum*	MHNL	6
unknown	*Bombuspomorum*	MHNL	12
unknown	*Colletesfodiens*	MHNL	1
Total			78
